# Repeated intracranial empyema following cranioplasty in a patient with atopic dermatitis: a case report

**DOI:** 10.1186/s13256-021-02898-z

**Published:** 2021-07-15

**Authors:** Shuhei Kubota, Masaaki Nemoto, Yuki Sakaeyama, Chie Nakada, Masataka Mikai, Yutaka Fuchinoue, Kosuke Kondo, Naoyuki Harada, Nobuo Sugo

**Affiliations:** Department of Neurosurgery (Omori), School of Medicine, Faculty of Medicine, Toho University, 6-11-1, Omori-nishi, Ota-Ku, Tokyo, 143-8541 Japan

**Keywords:** Atopic dermatitis, Cranioplasty, Surgical-site infection, *Staphylococcus aureus*

## Abstract

**Background:**

Atopic dermatitis is a chronic inflammatory skin disease associated with pruritus. Skin affected by atopic dermatitis not only shows a high percentage of *Staphylococcus aureus* colonization, but corneal barrier dysfunction is also known to occur. It is considered a risk factor for bacterial infections in various areas of the body. However, the relationship between atopic dermatitis and bacterial infection following neurological surgery has not yet been reported. Here, we present a case of atopic dermatitis in which the surgical site became infected twice and finally resolved only after the atopic dermatitis was treated.

**Case presentation:**

A 50-year-old Japanese woman with atopic dermatitis underwent cerebral aneurysm clipping to prevent impending rupture. Postoperatively, she developed repeated epidural empyema following titanium cranioplasty. As a result of atopic dermatitis treatment with oral antiallergy medicines and external heparinoids, postoperative infection was suppressed by using an absorbable plastic plate for cranioplasty. The patient’s postoperative course was uneventful for 16 months.

**Conclusions:**

Atopic dermatitis is likely to cause surgical-site infection in neurosurgical procedures, and the use of a metal implant could promote the development of surgical-site infection in patients with dermatitis.

## Introduction

Atopic dermatitis (AD) is a chronic inflammatory skin disease associated with pruritus. AD patients are likely to develop bacterial infections due to frequent *Staphylococcus aureus* colonization at a rate as high as 80–100% [1, 2] and corneal barrier dysfunction [2, 3].

Patients with AD are at a high risk of developing bacteremia [3, 4], which is considered a risk factor for surgical-site infection (SSI) in some areas of the body [6–9]. Previously, no relationship between AD and bacterial infection following neurosurgical procedures has been reported; however, in this study, we report the case of a 50-year-old woman with AD who developed repeated staphylococcal infections after cranioplasty. She was subsequently treated for AD, and cranioplasty was performed without a metal implant, and demonstrated a good postoperative course.

## Case presentation

**Fig. 1:**
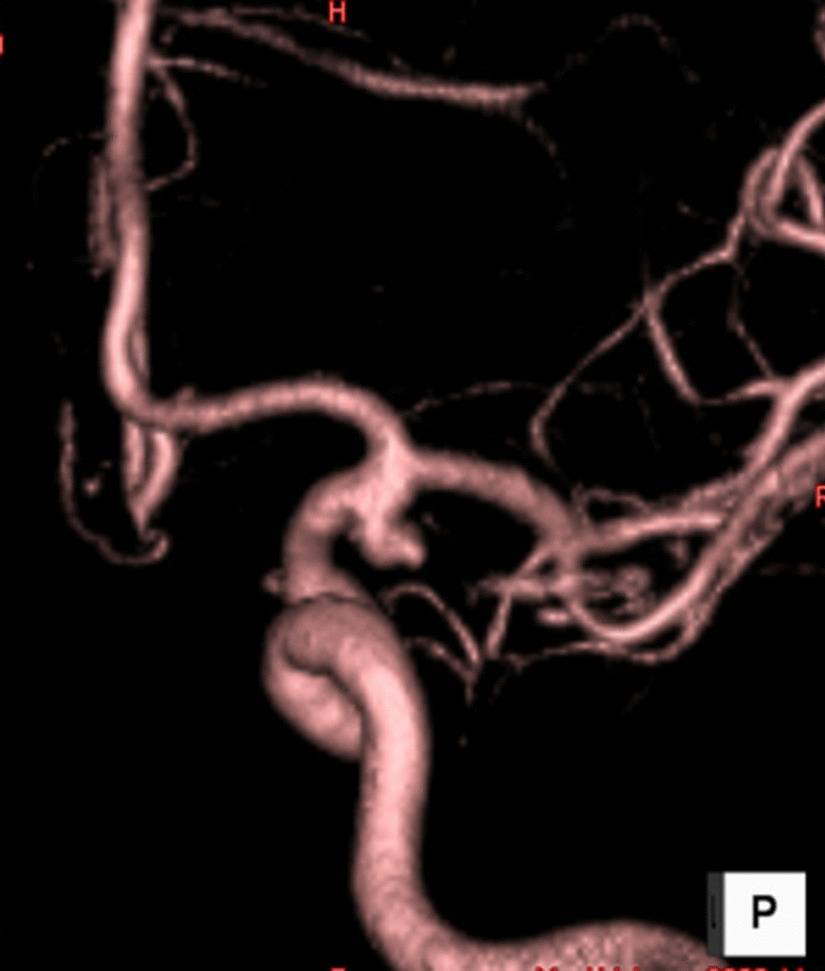
Three-dimensional image of cerebral angiography. Left internal carotid arterial angiography revealed a left internal carotid artery–anterior choroidal arterial aneurysm

A 50-year-old Japanese woman presented to us complaining of a sudden headache. Her medical history revealed she suffered from AD, and was allergic to pollen, house dust, acetaminophen, and gabapentin. A computed tomography (CT) scan of her head was performed when the patient first presented, showing no significant results. Digital subtraction angiography revealed a right internal carotid artery–anterior choroidal artery aneurysm (Fig. 1). Since impending rupture of the cerebral aneurysm was suspected, she underwent cerebral aneurysm clipping (Sugita Clip Titanium, MIZUHO Co., Tokyo, Japan) the following day, which was performed via right frontotemporal craniotomy. Titanium implants (Saney Plate system, Saney Seiko Inc., Saitama, Japan) and a pterion plate (Titanium skull mesh, Muranaka Medical Instruments Co., Osaka, Japan) were used in the cranioplasty. The postoperative course was uneventful, and she was discharged on postoperative day (POD) 13. On POD 25, she presented with swelling of the right eyelid and fever, at which point she was treated with oral cefazolin. However, she did not respond well to the treatment and was admitted to the hospital on postoperative day 33 because of continuous fever. On physical examination, eyelid swelling associated with tenderness was observed. She had a low-grade fever of 37.0 °C and eczema of the face. Meningeal signs were negative. Pale red papules, which are characteristic of AD, were observed on the face and fissures of both ear lobes. A blood test revealed that her white blood cell count (WBC) and C-reactive protein (CRP) levels were 7300/mm^3^ and 7.0 mg/dL, respectively. Based on these findings, the patient was hospitalized for SSIs.

**Fig. 2:**
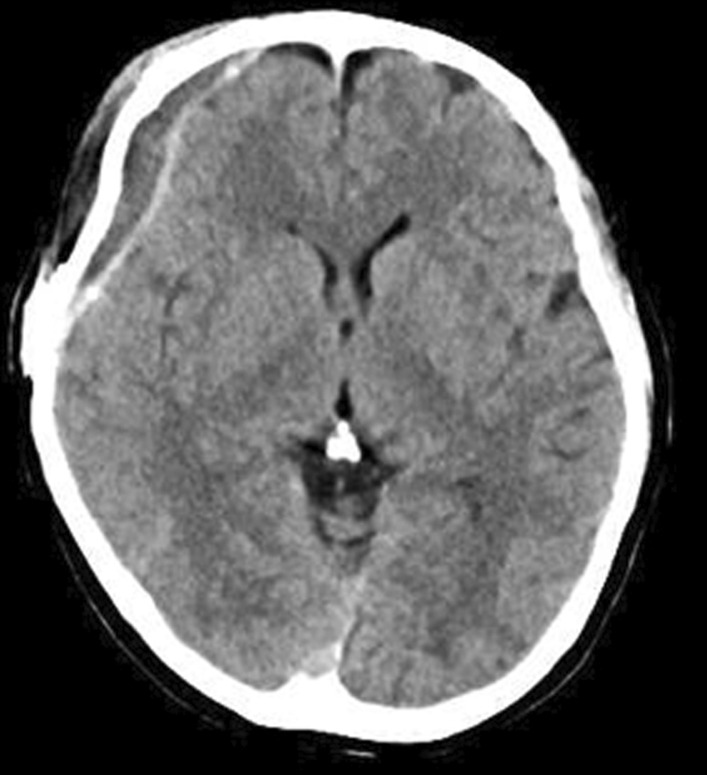
CT of first empyema. Isodense area of pooling was revealed in the right frontal epidural space

**Fig. 3:**
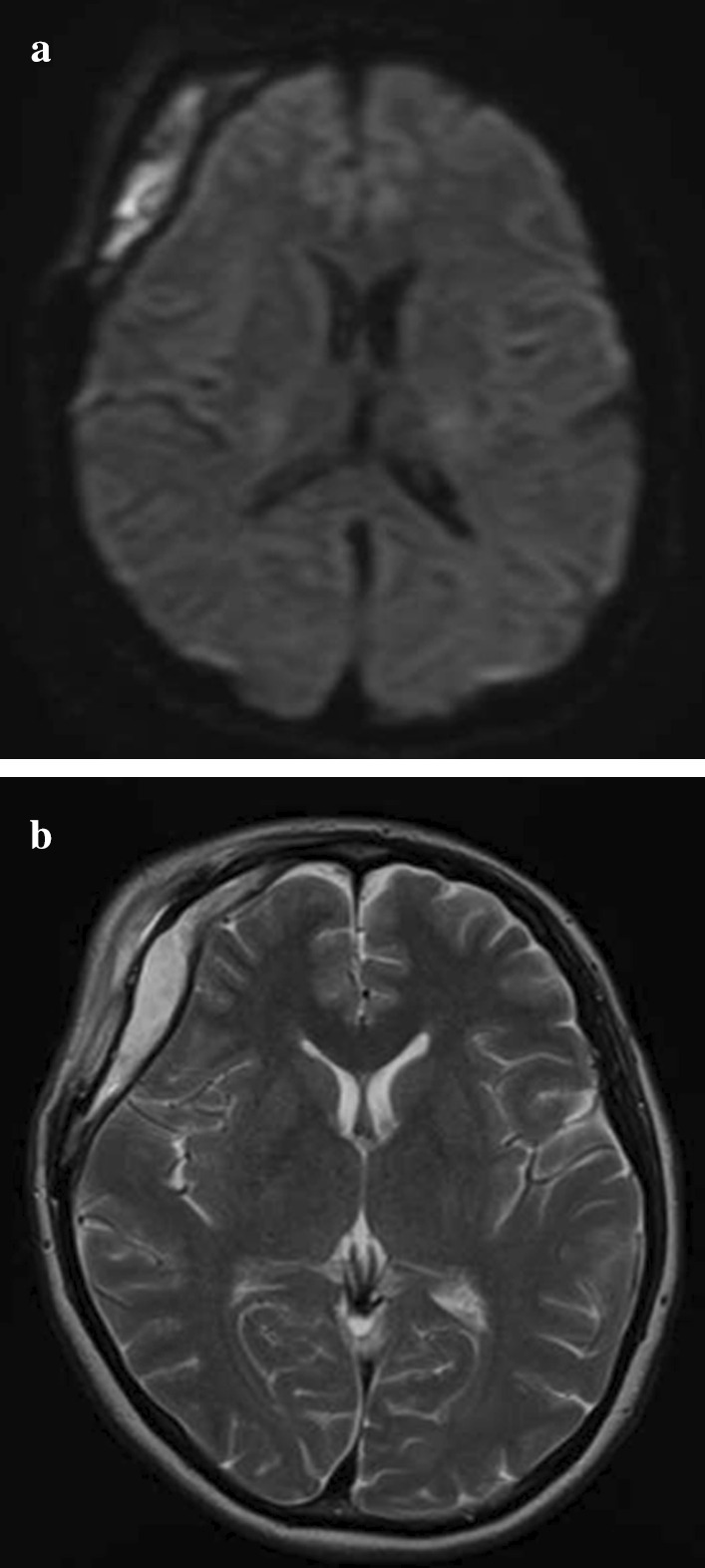
MRI of the first empyema. **a** Diffusion-weighted image. **b** T2-weighted image. The right frontal epidural area showed a high-intensity lesion on T2-weighted imaging and DWI

**Fig. 4:**
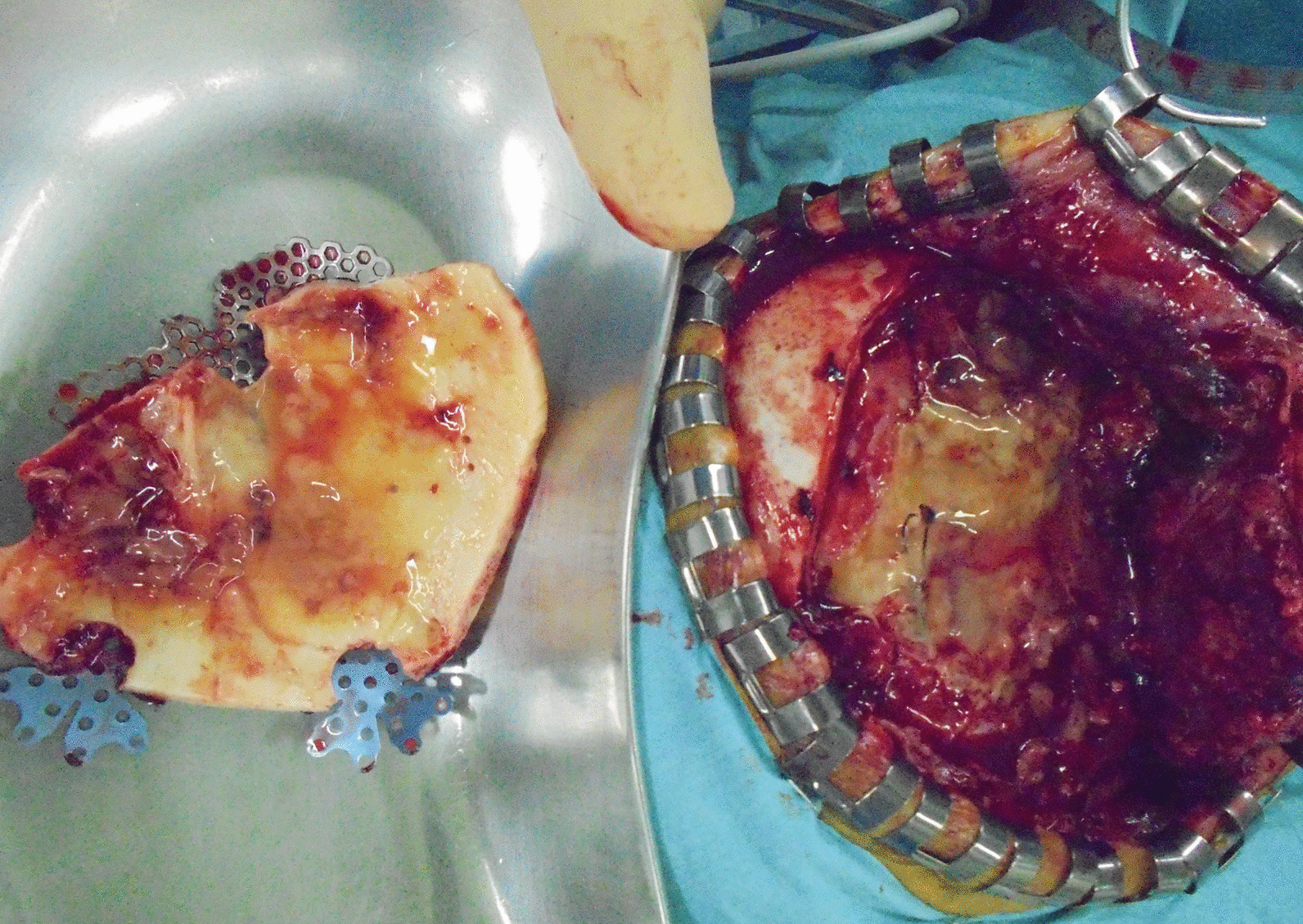
Intraoperative photograph of the first empyema. Pus was observed in the epidural space, while the dural surface was covered by granulation tissue

Neuroradiological findings were as follows: a head CT scan (Fig. 2) revealed isodense pooling in the right frontal epidural space. On magnetic resonance imaging (MRI), a corresponding high-intensity lesion was seen on diffusion-weighted imaging (DWI) (Fig. 3a) and on T2-weighted imaging (Fig. 3b). Epidural empyema due to SSI was suspected; therefore, she underwent emergent removal of the infected bone flap the day after admission. During surgery, pus was found in the epidural space, requiring irrigation with a high volume of saline (Fig. 4). After surgery, she was treated with intravenous meropenem at a dosage of 6 g/day. The culture revealed methicillin-sensitive *Staphylococcus aureus* (MSSA); thus, the antibiotic regimen was changed to cefazolin at a dosage of 6 g/day. Swelling of the eyelid and fever improved immediately after surgery, and both CRP and WBC levels decreased to within the normal limits; therefore, she was discharged 15 days after bone flap removal. Five months after the first bone flap removal, the patient underwent a second cranioplasty. In this procedure, artificial bone (ARTBONE, AHEAD Laboratories, CA, USA) was fixed using titanium plates (Saney Plate system, Saney Seiko Inc., Saitama, Japan). The patient was discharged 10 days after the operation without any signs of infection. Twenty-five days after the cranioplasty, the patient presented again with fever associated with headache and swelling of the right eyelid. The implant was removed because recurrent SSI was suspected. The operative findings were similar to those observed when the bone flap was removed. The muscle and subcutaneous tissue were swollen, and it was difficult to turn the skin flap. The dural surface was covered with a dark-red hematoma and yellow granulation tissue. These areas were irrigated as extensively as possible. The pus culture revealed MSSA. Intravenous cefazolin was initiated as antibacterial treatment. At this point, the AD was not cured. We also considered metal hypersensitivity as a differential diagnosis, and subsequently, she underwent a metal patch test. The test showed negative results for the metal samples, including titanium. She was also treated with oral levocetirizine dihydrochloride, (tranilast) at a dosage of 100 mg/day (two capsules per day), and a topical heparinoid. The dermatitis and pruritus have resolved. Fourteen months after the second implant removal, the patient underwent cranioplasty. The artificial bone (ARTBONE) was placed with an absorbable plastic plate (CRANIOFIX absorbable, B-BRAUN, Hessen, Germany). She has not developed another recurrent infection over 28 months following the last cranioplasty.

## Discussion

AD is likely to cause surgical-site infection (SSI) for two reasons: high colonization rate of *Staphylococcus aureus* on the skin and abnormal barrier function. Generally, patients with dermatitis have a high proportion of *Staphylococcus aureus* on their skin, whereas *Staphylococcus aureus* is not detected in normal, healthy skin. It has been reported that the detection rate of *Staphylococcus aureus* is approximately 80–100% in dermatitis lesions and 75% in non-dermatitis lesions [1, 2]. The severity of dermatitis is believed to be proportional to the detection rate of *Staphylococcus aureus* [1]. The barrier function of the stratum corneum is impaired by lipid reduction, and it has been demonstrated that scratching also stimulates bacteria that colonize the skin to invade the bloodstream [3, 4].

In this case, AD was suspected to be the cause of SSI because the first two cranioplasties, after which an infection developed, were performed before the AD was treated, while the cranioplasty after the AD was resolved resulted in no complications. To the best of our knowledge, there are no reports suggesting a relationship between AD and intracranial infection in neurosurgery. AD is reportedly related to SSI in other surgical specialties. Aoyagi *et al*. reported two cases of infectious endocarditis associated with AD, in which blood culture and skin colonization revealed *Staphylococcus aureus* of the same strain [5]. There are also some reports suggesting that AD increases SSI in ophthalmic and orthopedic surgeries [8–12]. The authors of these reports suspect that the high incidence of *Staphylococcus aureus* colonization in dermatitis is an important cause of postoperative infection. We believe that this would also increase the risk of infection after neurosurgical operations.

In general, sufficient treatment is needed to prevent postoperative infections in patients with AD, and it is necessary to plan the timing of surgery according to the condition of the skin [4]. During surgery, the dermatitis lesions must be completely drained. If they are not, the area should be washed well with povidone iodine. Blood contamination and stress due to gauze coverage may exacerbate the skin lesions. Hair washing as soon as possible may help reduce stress and prevent infection [4].

According to a survey of 348 cases reported by Tol *et al*., complications of cranioplasty include infections, which occur in 26.4% of patients, followed by convulsions (14.4%) and hematoma formation (6.9%) [13]. Other factors that could cause SSI after cranioplasty include age, hemorrhagic diseases, bifrontal cranioplasty, reoperation, long skin incision, long operation time, and thin connective tissue at the surgical site [13].

In this case, the contribution of using bioabsorbable implants instead of a titanium plate for preventing repeated infection is negligible. Metal implants may result in repeated infections. Generally, bioabsorbable plates are expected to reduce the risk of long-term infection. However, in the short term, some infections have been reported in cases using bioabsorbable implants [14, 15]. Considering that this patient had repeated infection in the short term, the effect of using bioabsorbable plate instead of titanium plate on recurrent infection may be insignificant.

## Conclusion

We encountered a case of AD complicated by repeated epidural abscesses after cranioplasty. In this case, sufficient control of the AD resulted in a good postoperative course. Preoperative treatment of AD after neurosurgical procedures should focus on the prevention of postoperative infections.

## Data Availability

All data generated or analyzed during this study are included in this published article.
